# The Army Dentist

**Published:** 1902-03

**Authors:** 


### THE ARMY DENTIST.

"From the paper of the president of the Army Dental Board, published in the *Dental Digest* for November, it appears that twenty-seven of the posts have been filled and that three positions are vacant. We presume that the dental societies of this country will be glad to welcome the occupants of these posts to membership in the societies adjacent to the army posts. So far as Chicago is concerned, the membership has been proffered to Dr. W. C. Fischer, who is located at Fort Sheridan. We had hoped that all dental societies would receive the army dental surgeon as a member, as soon as he was appointed, with open arms. At the last meeting of the Chicago Dental Society a resolution was passed giving the dental surgeon at Fort Sheridan all the privileges of membership to Dr. Fischer, but not providing for his successor in case he was transferred to some other post. We think that the least a society can do is to extend the courtesy of membership to a dental surgeon without dues, as he is liable to be sent elsewhere on short notice, and it seems only fair that he should be received with open arms by his compeers.

"For some reason best known to the army of objectors this was denied at the late meeting of the leading local society of Chicago by the usual objectors to all forms of real courtesy."—*Dental Review,* December 15, 1901.

The foregoing, from the *Dental Review,* exhibits a very liberal spirit, and, probably, will receive general approbation; in fact, it has already been strongly endorsed by at least one of the influential dental journals.

It is not pleasant to feel forced to dissent from the opinions expressed, but the policy outlined for dental societies to pursue in their relations with the young contract dental surgeons of the army is certainly a vicious one, and, if adopted, will lead to mischievous results.

These young men should be above receiving membership in any society upon the terms mentioned. The argument used, that they are liable to be moved from place to place, has no force; indeed, it is one reason why they should not be made members. If moved to various localities, they will have eventually a string of associations attached to their names, and in which they have taken, probably, no part or contributed either in money or ideas.

If these young men were without means, as many other young men are when beginning professional life, it would be a reasonable charity to aid them; but these army dentists are well paid in point of salary, and are able to add to this by work in after hours. They are, therefore, abundantly able to pay the yearly dues, and when forced to move should resign.

In view, however, of the possibility of removal, the generous and very proper course would be for the society nearest their place of work to invite them to attend the meetings, with all the privileges of the floor. This is all that should be given or should be required.

There should be a protest raised against making these young men special pets of the dental profession, and against the effort made in certain quarters to infuse into their minds that because they are dental surgeons in the army they are thereby placed in a class one degree better than the young graduate still struggling for an income to meet the ever-increasing expense account.

It is a matter for regret that the president of the Army Dental Examining Board has publicly endorsed the opinion of the *Dental Review.* The true position of an officer in the service should be a dignified opposition to all pecuniary favors, no matter in what form they may be presented.

